# Increased Lipolysis and Energy Expenditure in a Mouse Model with Severely Impaired Glucagon Secretion

**DOI:** 10.1371/journal.pone.0026671

**Published:** 2011-10-27

**Authors:** Phing-How Lou, Natalia Gustavsson, Yue Wang, George K. Radda, Weiping Han

**Affiliations:** 1 Laboratory of Metabolic Medicine, Singapore Bioimaging Consortium, Agency for Science, Technology and Research (A*STAR), Singapore, Singapore; 2 Department of Anatomy, Yong Loo Lin School of Medicine, National University of Singapore, Singapore, Singapore; 3 Department of Biochemistry, Yong Loo Lin School of Medicine, National University of Singapore, Singapore, Singapore; University of Hong Kong, China

## Abstract

**Background:**

Secretion of insulin and glucagon is triggered by elevated intracellular calcium levels. Although the precise mechanism by which the calcium signal is coupled to insulin and glucagon granule exocytosis is unclear, synaptotagmin-7 has been shown to be a positive regulator of calcium-dependent insulin and glucagon secretion, and may function as a calcium sensor for insulin and glucagon granule exocytosis. Deletion of synaptotagmin-7 leads to impaired glucose-stimulated insulin secretion and nearly abolished Ca^2+^-dependent glucagon secretion in mice. Under non-stressed resting state, however, synaptotagmin-7 KO mice exhibit normal insulin level but severely reduced glucagon level.

**Methodology/Principal Findings:**

We studied energy expenditure and metabolism in synaptotagmin-7 KO and control mice using indirect calorimetry and biochemical techniques. Synaptotagmin-7 KO mice had lower body weight and body fat content, and exhibited higher oxygen consumption and basal metabolic rate. Respiratory exchange ratio (RER) was lower in synaptotagmin-7 KO mice, suggesting an increased use of lipid in their energy production. Consistent with lower RER, gene expression profiles suggest enhanced lipolysis and increased capacity for fatty acid transport and oxidation in synaptotagmin-7 KO mice. Furthermore, expression of uncoupling protein 3 (UCP3) in skeletal muscle was approximately doubled in the KO mice compared with control mice.

**Conclusions:**

These results show that the lean phenotype in synaptotagmin-7 KO mice was mostly attributed to increased lipolysis and energy expenditure, and suggest that reduced glucagon level may have broad influence on the overall metabolism in the mouse model.

## Introduction

Glucose homeostasis is tightly controlled by two counter-acting hormones from the pancreas: insulin from beta-cells and glucagon from alpha-cells. Insulin promotes glucose clearance from the blood when blood glucose level is high, while glucagon restores normal blood glucose level during hypoglycemia by stimulating gluconeogenesis and glycogenolysis. The commonly accepted model attributes the development of type 2 diabetes (T2D) to relative insulin deficiency, which results from peripheral insulin resistance, and reduced insulin release due to defective insulin secretion and diminished beta cell mass [1]. Although much less mentioned, dysregulated glucagon secretion is another key factor that promotes hyperglycemia and diabetes development: loss of glucose inhibition of glucagon secretion leads to increased glucose production from the liver even in the presence of hyperglycemia [2].

Secretion of endocrine hormones, such as insulin and glucagon, is a highly regulated process and triggered by elevated intracellular calcium levels [3]. In the case of insulin, the increased calcium level is the result of a series of metabolic and membrane events that include increased ATP production, closure of ATP-sensitive potassium channels, membrane depolarization and opening of voltage-gated calcium channels [4]. The events preceding glucagon secretion are not as well defined, although it appears that ATP-sensitive potassium channels and voltage-gated calcium channels are also involved [5]. Another common feature between insulin and glucagon secretion is that they both require a calcium-binding protein, synaptotagmin-7, as the calcium sensor for insulin and glucagon granule exocytosis [3], [4], [6].

Synaptotagmin-7 belongs to a type-1 membrane protein family of more than 16 members [3], [7]. Synaptotagmins are characterized by an N-terminal transmembrane domain, a variable linker and two C-terminal C_2_ domains [8]. The C_2_ domains are the functional units of synaptotagmins, forming the basis for the function of synaptotagmins as calcium sensors in the regulation of exocytosis [3]. Deletion of synaptotagmin-7 in mice results in impaired insulin secretion when the mice are challenged by high glucose injection [4], and nearly abolished glucagon release at low blood glucose levels, such as when induced by insulin injection [6].

While the treatment and management of hyperglycemia especially in T2D has always been based on insulin response, emerging evidence suggests that inhibition of glucagon signalling presents a potential approach to the maintenance of normal glucose levels. Various pharmacological and genetic approaches have been used to evaluate the glycemic advantage of reduced glucagon production or signalling: (i) blocking glucagon signalling by targeted glucagon receptor gene deletion [9], [10], [11]; (ii) removal of endogenous glucagon using high affinity glucagon-neutralizing antibodies [12], [13], [14]; (iii) treatment with glucagon receptor anti-sense oligonucleotide [15], [16] or glucagon receptor antagonists [17], [18], [19], [20]; (iv) suppression of glucagon production via pancreas-specific ablation of the alpha-cell transcription factor, Arx, which results in a complete loss of the glucagon producing alpha-cells [21]. These studies collectively confirmed that disruption of glucagon production or signalling leads to an improvement in glucose metabolism in the animal models tested.

Consistent with the notion, our recent study showed that in the absence of dysregulated glucagon secretion, the combination of defective insulin secretion and peripheral insulin resistance was not sufficient to induce hyperglycemia in synaptotagmin-7 KO mice [1]. Despite impaired calcium-dependent insulin and glucagon granule exocytosis, synaptotagmin-7 KO mice maintain normal resting insulin level, but significantly reduced resting glucagon level [4], [6]. Furthermore, synaptotagmin-7 KO mice have lower body weight and body fat content [4]. In this study, we investigated the energy balance and regulation in the KO mice.

## Materials and Methods

### Ethics statement

All animal experiments in this study were conducted in accordance with the guidelines for animal care and use established by the Institutional Animal Care and Use Committee of the Agency for Science, Technology and Research (A*STAR) Biomedical Science Institutes in Singapore. The approved protocol numbers for the current study were IACUC #080351 and #090428.

### Animal welfare

Synaptotagmin-7 knockout (KO) mice were generated as previously described [22]. Only male mice were used in this study, and they were bred and housed in our animal facility. They were maintained at 24±1°C on a 12 h/ h light/dark cycle (7:00–19:00 h), and allowed free access to water and standard rodent chow (Harlan 2018 Teklad Global 18% Protein Rodent Diet).

### Body composition and core body temperature measurements

Age-matched littermates of synaptotagmin-7 KO and control mice were weighed, and their body composition was measured at 24 weeks of age by using an EchoMRI-100 (Echo Medical Systems) essentially as previously described [4]. Mice were anesthetised by using isoflurane before measurement of rectal temperature.

### Indirect calorimetry

Oxygen consumption (VO_2_), carbon dioxide production (VCO_2_), respiratory exchange ratio (RER), physical activity, and food intake were simultaneously measured for each mouse after a 1-day acclimatization period by using the Oxymax/Comprehensive Lab Animal Monitoring System (Columbus Instruments, Ohio) as previously described [23]. All oxygen consumption measurements were normalized to lean mass (ml/hour/kg lean mass) since lean tissues contribute more to the total energy expenditure compared to fat mass [24]. RER, a measure of metabolism substrate choice (carbohydrate or fat), was calculated as the ratio between VCO_2_ and VO_2_. All data collected were averaged over a monitoring period of 6 days.

### Plasma hormone and analyte measurements

Blood samples were collected from tail vein for glucose measurements by using Accu-Chek Advantage glucometer (Roche). Plasma samples were prepared by blood centrifugation at 8,000 x g for 5 min at 4°C. Mouse Insulin ELISA Assay (Mercodia), Glucagon RIA (Millipore), Mouse Leptin ELISA (Millipore) were used for plasma insulin, glucagon, and leptin measurements, respectively. Non-esterified fatty acids (NEFA) were measured with a Wako kit. Triglyceride levels were determined in blood with Accutrend GCT System (Roche).

### Tissue analysis of ATP and lipid content

Mice were killed by cervical dislocation and their hind limbs were snap-frozen in liquid nitrogen within 1 second. Gastrocnemius muscle was dissected free of visible fat and connective tissues on a metal plate that was cooled by liquid nitrogen. Muscle samples were then homogenised in lysis buffer (supplied in ATP Bioluminescence Assay Kit HS II, Roche). After centrifugation at 16,000 x g for 10 min at 4°C, 50 µl of supernatant was assayed for ATP by using ATP Bioluminescence Assay Kit HS II (Roche).

Extraction of lipid content in skeletal muscle was performed as previously described [25]. Briefly, skeletal muscle tissue was homogenized with chloroform/methanol (2∶1) at sample/solvent ratio of 1∶5, and then re-homogenized with 1 part chloroform. One part distilled H_2_O was then added and the homogenate was vortexed before centrifugation at 350 x g to separate the aqueous phase from the organic phase. The lower organic phase was carefully recovered for triglyceride and glycerol content analysis by using Serum Triglyceride Determination Kit (Sigma).

### Mitochondrial isolation from skeletal muscle

Mitochondrial isolations were performed on groups of synaptotagmin-7 KO and control mice at 24 weeks of age. Mice were killed by cervical dislocation. Skeletal muscle was rapidly dissected from both hind limbs, cleaned of fat and connective tissues, and collected in ice-cold medium comprising 100 mM KCl, 50 mM Tris/HCl, and 2 mM EGTA (pH 7.4 at 4°C). Mitochondria from skeletal muscle were isolated essentially as previously described [26], but with defatted BSA. Mitochondrial protein concentration was determined by the Bradford method, with BSA as the standard.

### Western blotting

Samples of mitochondria or tissue lysate were solubilized and resolved on 10% or 12.5% SDS/PAGE gel. Proteins were blotted onto PVDF membrane and probed using the following antibodies: UCP1, CPT1 (Santa Cruz Biotechnology), UCP3 (Abcam), HSL, pHSL (Cell Signaling), and beta-tubulin (Sigma).

### Statistical analysis

Data are presented as means ±SEM. Comparisons of data were made by using two-tailed Student's t-test or ANOVA followed by Dunnett's post hoc test. Statistical significance is displayed as *p<0.05 or **p<0.01.

## Results

### Higher metabolic rate in synaptotagmin-7 KO mice

**Figure 1:**
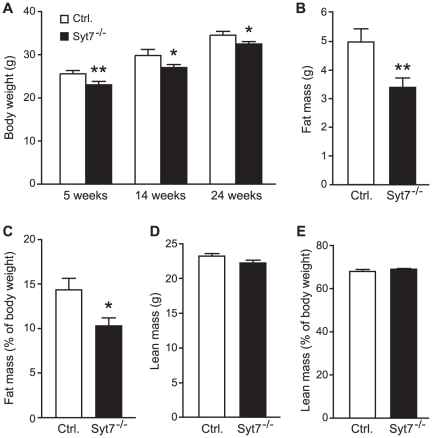
Reduced body weight and body fat mass in synaptotagmin-7 KO mice. (A) Body weights of synaptotagmin-7 KO (Syt7^-/-^) and control (Ctrl.) mice at 5, 14, and 24 weeks of age. N = 8–32 per group. (B, C) Fat mass (B) and proportion of body fat (C) in synaptotagmin-7 KO and control mice at 24 weeks of age. (D, E) Lean mass (D) and proportion of body lean (E) in synaptotagmin-7 KO and control mice at 24 weeks of age. All data are expressed as means ± SEM. N = 24–32 per group. *, P<0.05; **, P<0.01.

Although deletion of synaptotagmin-7 results in 40–50% reduction of insulin secretion and nearly abolished glucagon secretion, the synaptotagmin-7 KO mice are generally normal under non-stressed conditions. One distinctive phenotype was that synaptotagmin-7 KO mice exhibited lower body weight throughout the monitoring period from 5 to 24 weeks when compared with the control mice (Figure 1A).

Body composition analysis by MRI revealed lower total body fat in the 24-week old synaptotagmin-7 KO mice compared to the control mice (Figure 1B). The proportion of body mass in fat was also significantly lower in the synaptotagmin-7 KO mice (Figure 1C). No significant difference in lean body mass was observed (Figure 1D-E). Although there were no apparent abnormalities in adipose tissue, adipocytes from synaptotagmin-7 KO mice appeared to be slightly smaller (Figure S1). These results indicate that the lower body weight of the synaptotagmin-7 KO mice could be attributed to their lower fat content.

**Figure 2:**
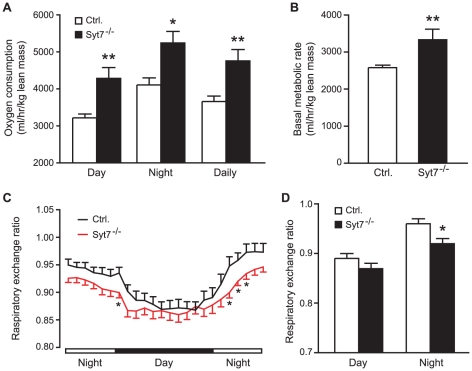
Enhanced metabolism in synaptotagmin-7 KO mice. (A) Rate of daily oxygen consumption for synaptotagmin-7 KO (Syt7^-/-^) and control (Ctrl.) mice during the day, night and whole-day periods. (B) Basal metabolic rate as measured in terms of oxygen consumption (ml/hour/kg lean mass) for synaptotagmin-7 KO and control mice. (C) RER of synaptotagmin-7 KO and control mice over a 24-hour monitoring period. (D) RER calculated from data in (C) during day (0700–1900 h) and night (1900–0700 h). All data are presented as means ± SEM. N = 9–10 per genotype. *, P<0.05; **, P<0.01.

To test whether the lower body weight phenotype in synaptotagmin-7 KO mice was due to reduced food intake and/or increased energy expenditure, we performed indirect calorimetry on synaptotagmin-7 KO and control mice. Food intake, either in absolute amount or when normalized to body weight, showed no difference between the two genotypes (3.79±0.15 g/day, N = 9 for synaptotagmin-7 KO mice; 3.67±0.25 g/day, N = 10 for control mice). We then tested energy expenditure in synaptotagmin-7 KO and control mice. Oxygen consumption during the day and night periods was higher in the synaptotagmin-7 KO mice (Figure 2A). Locomotor activity was indistinguishable between control (56,164±6,263 counts per day, N = 9) and synaptotagmin-7 KO mice (63,883±6,008 counts per day, N = 10). Furthermore, basal metabolic rate (based on the rate of resting VO_2_) was approximately 27% higher in the synaptotagmin-7 KO mice (Figure 2B). A similar trend of higher carbon dioxide production (VCO_2_) was also observed in the synaptotagmin-7 KO mice (data not shown). These data show that the synaptotagmin-7 KO mice had increased whole body metabolism. During the night period when mice are active, synaptotagmin-7 KO mice maintained lower RER than the control mice (Figure 2C and 2D), suggesting that the synaptotagmin-7 KO mice consumed a higher percentage of fat in their energy production than control mice.

### Enhanced lipolysis in synaptotagmin-7 KO mice

**Figure 3:**
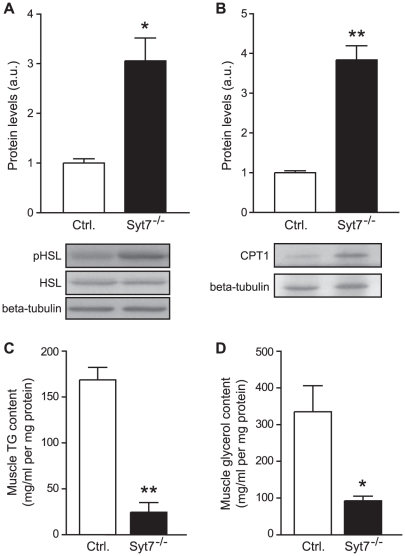
Enhanced lipolytic enzyme and reduced muscle TG content in synaptotagmin-7 KO mice. (A, B) Representative immunoblots and quantification of protein levels of phopho-HSL (pHSL) from fat pads (A), and CPT1 from skeletal muscle mitochondria (B) of 20–24 week old synaptotagmin-7 KO and littermate control mice. Densitometric quantification for each protein was normalized against beta-tubulin from 3-5 independent pairs of synaptotagmin-7 KO and control mouse samples. (C) Triglyceride (TG) and (D) glycerol content from muscle samples. N = 4 per genotype. Data are presented as means ± SEM. *, P<0.05; **, P<0.01.

Considering that synaptotagmin-7 KO mice consumed more fat as fuel than the control mice, we tested whether synaptotagmin-7 KO mice had increased lipolysis and fat oxidation. Hormone-sensitive lipase (HSL) catalyses the hydrolysis of triacylglycerol, the rate-limiting step in lipolysis, and is the key enzyme in the regulation of lipid stores. Several mechanisms are known to regulate the activity of HSL, one of which is via the reversible phosphorylation of HSL by protein kinases [27]. HSL activity is acutely stimulated when protein kinase A (PKA) phosphorylates HSL at Ser563, Ser659 and Ser660 [28], [29]. Densitometric analysis of pHSL (Ser660) revealed that the activity of HSL per unit of adipose tissue isolated from synaptotagmin-7 KO mice was increased 2-fold (Figure 3A), consistent with increased lipolysis in synaptotagmin-7 KO mice.

Enhanced lipolysis is usually accompanied by increased plasma triglycerides (TG) and free fatty acids (FFA) levels. However, we found reduced TG and FFA levels in synaptotagmin-7 KO mice at resting state (Table 1). This suggests increased uptake and/or oxidation of fatty acids in muscle cells, as skeletal muscle is a major site of fatty acid oxidation [30]. Carnitine palmitoyltransferase I (CPT I) is the principal modulator of fatty acid oxidation (beta-oxidation) since it catalyses the transport of fatty acyl-CoA across the mitochondrial membrane, and thus controls the fatty acid flux through beta-oxidation [30]. Over-expression of CPT I protein alone has been demonstrated to increase fatty acid oxidation in both isolated mitochondria and intact skeletal muscle [31]. So we examined the level of CPT I expression in skeletal muscle mitochondria isolated from both groups of mice as an indirect gauge of fatty acid oxidation. CPT I protein level was almost 3-fold higher in the synaptotagmin-7 KO mice than in the control mice (Figure 3B), supporting the notion that fatty acid oxidation was higher in synaptotagmin-7 KO mice.

**Table 1:** Plasma or serum analytes of synaptotagmin-7 KO and control mice.

	Control			Synaptotagmin-7 KO		Statistics
	Mean ± SEM	No.		Mean ± SEM	No.	
Glucose (mM)						
Fed	8.7±0.3	13		8.8±0.3	13	NS
Fasting	5.5±0.2	13		5.1±0.2	13	NS
Insulin (ng/ml)						
Fed	4.08±0.46	13		3.31±0.28	13	NS
Fasting	1.35±0.08	13		1.02±0.09	13	P<0.05
Glucagon (pM)						
Fed	83.0±9.2	13		51.6±5.3	13	P<0.01
Fasting	116.5±6.9	12		53.7±6. 7	12	P<0.001
Lipids						
Cholesterol (mM)	4.13±0.02	7		4.10±0.02	6	NS
Triglycerides (mM)	1.80±0.19	7		1.25±0.07	6	P<0.05
Free fatty acids (µM)	253.1±24.8	13		170.0±20.7	13	P<0.01

Intramuscular TG and glycerol content represents the balance of fat accumulation and oxidation in the muscle. We found that synaptotagmin-7 KO mice showed reduced TG and glycerol in muscle, consistent with increased fat combustion (Figure 3C and 3D). Lipolysis yields more ATP than carbohydrate catabolism and increased usage of fat in energy production may be reflected by ATP content in muscle. Synaptotagmin-7 KO mice had higher ATP level than control mice (46.2±4.9 versus 34.0±0.3 nmol/mg protein, synaptotagmin-7 KO and control, N = 7–8 per group; P<0.05). This is in agreement with the hypothesis that synaptotagmin-7 KO mice consumed a higher percentage of fat in energy production based on indirect calorimetry analysis.

Consistent with higher energy production, the core body temperature was higher in synaptotagmin-7 KO mice than the control group (38.0±0.2 versus 37.4±0.2°C, synaptotagmin-7 KO and control, N = 10–12 per group; P<0.05). To test whether the increased temperature was associated with inflammation, we measured several pro-inflammatory cytokines in both groups of mice, including IL1-α, IL1-β and IL-6, but found no difference (data not shown).

### Upregulated expression of UCP3 in skeletal muscle mitochondria of synaptotagmin-7 KO mice

**Figure 4:**
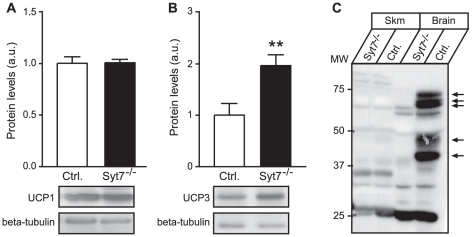
Increased UCP3 expression in the skeletal muscle mitochondria of synaptotagmin-7 KO mice. (A, B) Representative immunoblots of UCP1 from brown adipose tissue (A) and UCP3 from skeletal muscle (B) of 20–24 week old synaptotagmin-7 KO and control mice. Relative densitometric quantification was normalized against beta-tubulin. Each set of samples was from 3–5 mice. (C) Synaptotagmin-7 (arrows) was not detected in skeletal muscle. Data are presented as means ± SEM. **, P<0.01.

The observed lower body weight and body fat, coupled with increased metabolic rate and core body temperature, suggest increased uncoupling protein (UCP) activity. We examined the expression levels of UCP1 and UCP3. UCP1 is exclusively found in brown adipose tissue and is known to mediate facultative thermogenesis in hibernating mammals and rodents [32]. UCP1 expression in the brown adipose tissue of synaptotagmin-7 KO mice was similar to that of the control mice (Figure 4A), whereas the expression of UCP3 was significantly elevated in the skeletal muscle of synaptotagmin-7 KO mice (Figure 4B). In adult mice, synaptotagmin-7 expression was mostly restricted to brain and endocrine cells [33], and was not detected in skeletal muscle (Figure 4C). Thus, it is unlikely that the up-regulation of UCP3 was a direct effect of synaptotagmin-7 deletion.

## Discussion

Glucagon restores normal blood glucose levels by increasing hepatic glucose production and disruption of glucagon signalling results in hypoglycemia [9], [10], [12], [13], [14], [15], [16], [17], [18], [19], [20]. In synaptotagmin-7 KO mice, calcium-dependent glucagon secretion was nearly abolished [6], and lower blood glucose was observed when synaptotagmin-7 KO mice were subjected to prolonged starving [1]. On the other hand, dysregulated glucagon secretion and enhanced glucagon signalling may directly promote hyperglycemia and diabetes [1], [2], [34]. We recently showed that without dysregulated glucagon secretion, the combination of defective insulin secretion and peripheral insulin resistance was not sufficient to induce hyperglycemia in synaptotagmin-7 KO mice [1]. In light of renewed interest in targeting glucagon signalling for the prevention and treatment of hyperglycemia and diabetes, it is important to understand how absolute glucagon deficiency and impaired glucagon secretion affect energy homeostasis.

Although deletion of synaptotagmin-7 results in impaired insulin secretion in response to high glucose challenge, synaptotagmin-7 KO mice exhibit normal resting insulin levels under non-stressed conditions when the mice have free access to feed ([4] and data not shown). In contrast to insulin, glucagon levels in synaptotagmin-7 KO mice are always lower, and do not increase after fasting (Table 1) [4], [6]. Therefore, synaptotagmin-7 KO mice may be used to understand the metabolic consequences of glucagon deficiency. Consistent with previous observations, synaptotagmin-7 KO mice have lower body weight and are leaner than the control mice [4] (Figure 1). Here we show that the lower body weight was not due to reduced food intake, but rather increased energy expenditure in synaptotagmin-7 KO mice as indicated by the higher oxygen consumption rate and higher basal metabolic rate (Figure 2A and 2B). Furthermore, metabolic analysis indicates that the higher energy expenditure could be largely attributed to increased use of fat as fuel in these mice (Figure 2C and 2D), as synaptotagmin-7 KO mice show upregulation of lipolytic enzyme (Figure 3A) and increased capacity for fatty acid transport and oxidation (Figure 3B-D). In addition, synaptotagmin-7 KO mice exhibit non-inflammatory hyperthermia due to mitochondrial uncoupling, possibly through increased UCP3 expression (Figure 4B), or increased fatty acid oxidation [35]. These metabolic alterations are contributing factors to the observed lean phenotype in the synaptotagmin-7 KO mice.

The mechanisms by which energy expenditure was increased through lipolysis are unclear. Our findings that synaptotagmin-7 KO mice have reduced lipid accumulation in skeletal muscle and low circulating levels of TG and FFA suggest that synaptotagmin-7 KO mice have developed compensatory mechanisms for the persistent low glucagon levels, for example by increasing lipolytic enzyme levels. Consistent with increased fatty acid oxidation, mitochondria isolated from skeletal muscles of synaptotagmin-7 KO mice showed increased UCP3 expression (Figure 4B). The link between increased UCP3 expression and enhanced fatty acid oxidation was also reported previously: (i) UCP3 expression was increased in animal and human subjects undergoing metabolic states with high fatty acid oxidation (fasting [36], acute bouts of exercise [37], [38], and high fat feeding [39]); (ii) increased fatty acid oxidation was observed in UCP3 over-expression mouse studies [40] or other over-expression systems [41]. The exact role of UCP3 in fatty acid oxidation is still unclear, but one proposed role for UCP3 is in the export of fatty acid anions thus permitting continuous rapid fatty acid oxidation during an oversupply [42]. It is also unclear how the increased lipolysis occurred in the synaptotagmin-7 KO mice. Sympathetic nerve activity and catecholamines are known triggers of increased lipolysis and UCP3 expression [43], [44]. Although deletion of synaptotagmin-7 impairs secretory granule exocytosis in chromaffin cells [45], we did not observe significant differences in epinephrine, norepinephrine or dopamine levels between synaptotagmin-7 KO and control mice (data not shown).

In summary, the present study reveals altered metabolic responses in synaptotagmin-7 KO mice, including increased lipolysis, fatty acid transport and oxidation, and the increased use of fat as fuel. The increased fatty acid oxidation or utilization observed in the present study provides a plausible explanation for the lean phenotype in the synaptotagmin-7 KO mice. This study, however, does not indicate that changes to metabolism are a direct effect of synaptotagmin-7 deletion but possibly the result of systemic effect of glucagon deficiency on the overall animal metabolism. Our study corroborates certain beneficial effects of reduced glucagon signalling, and supports its therapeutic potential in the prevention and treatment of diabetes and other metabolic-related diseases.

## Supporting Information

Figure S1 **Comparison of white adipose tissue from synaptotagmin-7 KO and control mice.**H&E staining of white adipose tissue sections from synaptotagmin-7 KO (Syt7^-/-^) and control (Ctrl.) mice. Images were obtained on a Nikon inverted microscope. Scale bar  = 200 µm.
(EPS)
